# Infrared and Raman spectroscopy of blood plasma for rapid endometrial cancer detection

**DOI:** 10.1038/s41416-025-03050-0

**Published:** 2025-05-18

**Authors:** Roberta Schiemer, Jessica Grant, Mohamad N. Shafiee, Sendy Phang, David Furniss, Radu Boitor, Angela B. Seddon, Ioan Notingher, William Atiomo, Nia W. Jones, Ketankumar B. Gajjar

**Affiliations:** 1 Division of Child Health, Obstetrics and Gynaecology, University of Nottingham, Nottingham, UK; 2 Mid-Infrared Photonics Group, George Green Institute for Electromagnetics Research, Faculty of Engineering, University of Nottingham, Nottingham, UK; 3 Department of Obstetrics and Gynaecology, Faculty of Medicine, Universiti Kebangsaan Malaysia, Cheras, Kuala Lumpur Malaysia; 4 School of Physics and Astronomy, University of Nottingham, Nottingham, UK; 5 College of Medicine, Mohammed Bin Rashid University of Medicine and Health Sciences (MBRU), Dubai, United Arab Emirates; 6 Division of Lifespan and Population Health, University of Nottingham, Nottingham, UK

**Keywords:** Endometrial cancer, Optical spectroscopy

## Abstract

**Background:**

Endometrial cancer (EC) is the 6th most common cancer among women worldwide. No effective non-invasive screening methods or approved blood biomarkers for EC exist. Previous research explored Attenuated Total Reflection-Fourier Transform Infrared (ATR-FtIR) and Raman spectroscopies, using dried blood plasma. Fresh, ‘wet’, blood samples, that might provide faster results, have not been investigated. This study compared ATR-FtIR and Raman spectroscopies on ‘wet’ and dry blood plasma samples for EC detection. It also conducted a preliminary exploration into their diagnostic potential for EC in high-risk individuals with polycystic ovary syndrome (PCOS).

**Methods:**

‘Wet’ and dry blood plasma samples from participants with EC, PCOS and healthy controls were analysed using ATR-FtIR and Raman spectroscopies. Machine learning algorithms and multivariate statistical analyses assessed spectral variance across datasets to evaluate the techniques’ diagnostic performance.

**Results:**

Raman analysis of ‘wet’ plasma achieved 82% accuracy in detecting EC, while ATR-FtIR spectroscopy reached 78%. When combined, diagnostic accuracy reached 86%. In comparison, dry plasma analysis with ATR-FtIR detected EC with 83% accuracy. Spectral similarities were found between EC and PCOS.

**Conclusions:**

Our study suggests that ATR-FtIR and Raman spectroscopies could revolutionise early diagnosis of EC. More research is required to validate these promising findings.

## Background

Endometrial cancer (EC) ranks as the 6th most common cancer in women worldwide, with over 417,000 new cases diagnosed in 2020 [1]. A 60% increase in EC incidence rates in the UK from the 1990s to 2018 [2] and the predicted 12% rise in mortality rates between 2023–2025 and 2038–2040 [3] highlight the growing EC disease burden.

Several risk factors have been identified for EC. The leading theory behind endometrial carcinogenesis points to an increased lifetime exposure to oestrogen, which stimulates endometrial proliferation [4]. This is a predominant feature in obesity and metabolic syndromes with insulin resistance/hyperinsulinaemia, such as polycystic ovary syndrome (PCOS) [4]. Individuals with PCOS are three times more likely to develop EC compared to individuals without PCOS, with the risk increasing to almost six times in pre-menopausal women [5]. Additionally, a genetic predisposition to EC is seen in individuals carrying mutations in mismatch repair genes (MLH1, MSH2, MSH6, PMS2), associated with Lynch syndrome [6].

EC has traditionally been classified into two main histological types, each associated with different sets of risk factors: Type I (endometrioid adenocarcinoma), the most common form, which is linked to unopposed oestrogen exposure, and Type II (non-endometrioid, including serous and clear cell carcinomas as well as squamous cell carcinoma and carcinosarcoma), which is non-oestrogen dependent and tends to display more aggressive behaviour, with a higher risk of extra-uterine disease at first presentation [7–9]. Recent advances in the genomic and proteomic characterisation of EC, particularly through The Cancer Genome Atlas (TCGA) project [10], have led to a refined understanding of its molecular landscape and the identification of four EC molecular subtypes. This has facilitated the integration of pathological and molecular classifications, providing more accurate prognostic information and enabling tailored treatment strategies. Approximately 67% of patients with EC present at early stage, where the prognosis is excellent [11]. In contrast, patients diagnosed with advanced disease have a 5-year survival rate of ~15% [12, 13]. In addition, diagnosing EC at stage I incurs total 5-year diagnostic and treatment costs that are almost three times lower than those for stage III diagnoses [13]. This highlights the significant benefits of early disease detection and screening, not only for improving patient outcomes but also for reducing healthcare costs [12, 13].

While endometrial surveillance is recommended for patients with Lynch syndrome, surveillance programmes for these patients vary across countries and centres, survival data are limited, and data are lacking on the impact of surveillance on mortality reduction [6]. Conversely, there are no established population-level EC surveillance programmes globally, nor for individuals with risk factors such as high body mass index (BMI) or PCOS. The assessment of endometrial thickness using transvaginal ultrasonography has been investigated as a non-invasive screening strategy [14]. However, the 5 mm cut-off for endometrial thickness has shown poor diagnostic accuracy for the screening of asymptomatic postmenopausal women, who would be the likely primary target of such programmes, alongside high-risk patients [15]. Endometrial biopsy is more effective, but it is an invasive procedure and this limits its application as a population-level screening tool [4, 16]. Several blood-based prognostic biomarkers have shown potential for risk stratification in EC, including proteins such as cancer antigen 125 (CA125) and Human Epididymis Protein 4 (HE4), as well as circulating cell-free tumour DNA levels [17, 18]. However, none of these biomarkers is currently approved for routine clinical use [19]. There is therefore an urgent need for research into less invasive, inexpensive diagnostic and screening techniques for EC.

A promising alternative approach to conventional EC diagnostic methods is the non-invasive approach of blood plasma analysis using vibrational biospectroscopy techniques. Rapid and non-destructive, ATR-FtIR and Raman spectroscopies utilise the absorption and inelastic scattering interactions, respectively, between incident light and chemical bonds within a biological sample [20–22]. Here, the wavelengths of light (photon energies) absorbed or inelastically scattered by a sample are dependent on the specific discrete ground state and fundamental vibrational frequencies of the chemical bonds present [23]. Spectral patterns of ATR-FtIR absorption bands and Raman scattering bands are thus unique to the biological sample, providing a digital ‘molecular bio-fingerprint’. This is subject to change upon alterations in the chemical bonds present, such as in the case of cancer development [24].

Considered complementary in nature, ATR-FtIR and Raman spectroscopies use a mid-infrared (MIR) and near-infrared (NIR)/visible light source, respectively. These two spectroscopic techniques feature differing quantum selection rules, whereby one technique enables interactions with certain chemical bonds undetected or less detected by the other [23]. Chemical bonds are considered infrared-active and detectable using ATR-FtIR spectroscopy when their absorption of incident light increases the amplitude of molecular vibration, sufficiently to induce a change in the bond dipole moment [22]. Conversely, chemical bonds are Raman active if incident radiation can excite them to a virtual state, causing a change in the molecule polarisability [22].

Blood samples are an attractive choice for spectroscopy analysis given the associated low procedural costs, the high patient acceptability and minimally invasive nature of sample collection, which makes them ideal candidates for screening test development.

Preliminary studies demonstrated promising results using ATR-FtIR spectroscopy with dried blood plasma samples for EC detection [25–28]. Indeed, the study of biofluids using spectroscopy is a rapidly advancing field, with potential applications spanning not only oncology but also a wide array of acute and chronic medical conditions [29–37].

The highly infrared (IR)-active nature of water molecules, with broad absorption bands, is commonly thought to conceal the spectral bands of important chemical bonds [38]. Thus, the use of dried blood plasma over *‘*wet’ samples has been conventionally exclusively favoured when utilising ATR-FtIR spectroscopy for cancer detection [25–28]. In the context of rapid diagnosis and development of EC screening, however, the use of fresh, ‘wet’, blood samples would be more advantageous. ‘Wet’ analysis would potentially increase speed and efficiency of sample processing, eliminating the additional time, resources and costs required for sample dehydration. Additionally, fresh analysis protocols could allow future development of tools for in vivo bedside diagnosis. An evaluation of ATR-FtIR spectroscopy diagnostic performance on ‘wet’ blood plasma samples has not previously been conducted for EC patients. Similarly, Raman spectroscopy has been used successfully for EC detection in biopsied endometrial tissue samples [39], and for detection of malignancies such as breast, cervical and oesophageal cancer [40–42]. Recent studies have also demonstrated its potential for non-invasive investigation of carcinogenesis, through the identification of markers associated with epithelial-mesenchymal transition (EMT) by means of surface-enhanced Raman spectroscopy (SERS) [43, 44]. However, Raman has not previously been applied to EC detection using ‘wet’ blood plasma samples.

In this study, we directly compare the efficacy of ATR-FtIR and Raman spectroscopies applied to both ‘wet’ and dry blood plasma for EC detection. Our primary aim is to determine whether one technique is superior or if their combination can synergistically enhance diagnostic performance. This evaluation is necessary to inform future developments in EC screening and diagnosis. We introduce a novel approach for processing spectral data from both techniques, enabling a comprehensive analysis of their combined performance. To our knowledge, this is the first study to utilise ‘wet’ alongside conventional dry blood plasma to assess the diagnostic capabilities of independent and combined ATR-FtIR and Raman spectroscopies for EC. Furthermore, this research conducted a preliminary exploration into their diagnostic potential for EC in high-risk individuals with PCOS, a context that has not previously been investigated.

## Materials and methods

### Study design

The primary aim of this study was to compare diagnostic abilities of ATR-FtIR and Raman spectroscopies using ‘wet’ and dry plasma, examining the spectroscopic techniques both independently and in combination. We also conducted a preliminary study of whether patients with PCOS exhibited any characteristic spectral pattern, more consistent with EC or with controls.

Blood plasma samples were obtained from a cross-sectional study conducted within the division of Lifespan and Population Health at Nottingham University Hospitals NHS (National Health Service) Trust in the United Kingdom. Our study population comprised 54 participants, 22 patients with EC and 32 controls. The control group consisted of 14 healthy participants and 18 participants with PCOS. The sample size was set at 54 participants as sample sizes between 24 and 50 are usually recommended for single-centre feasibility studies [45–47].

**Fig. 1:**
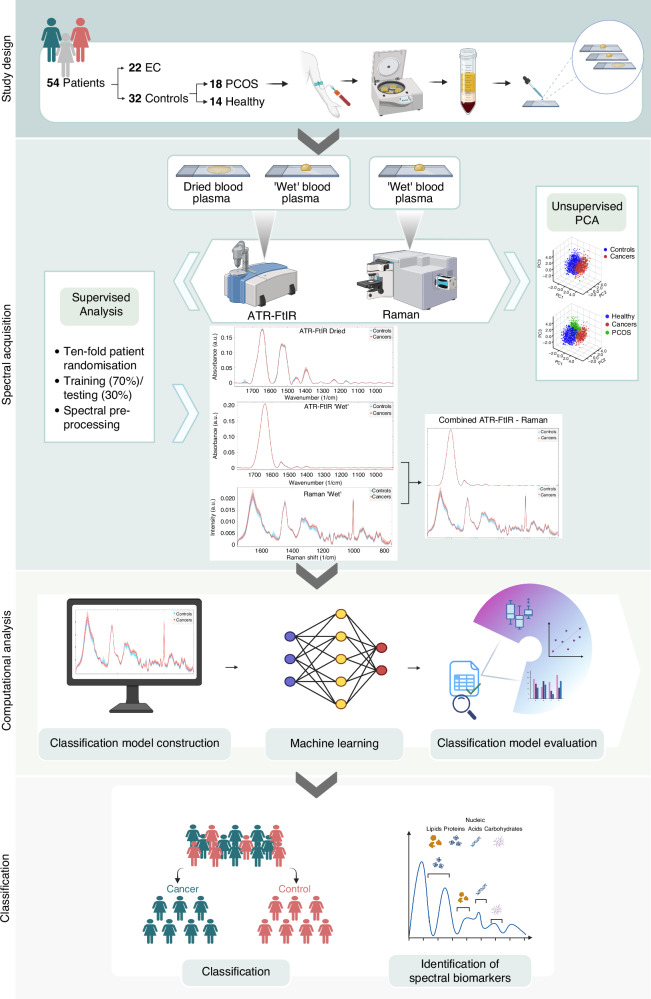
Concept map of the study. EC endometrial cancer, ATR-FtIR Attenuated Total Reflection-Fourier Transform Infrared, PCA principal component analysis, PCOS polycystic ovary syndrome. Created in BioRender: https://BioRender.com/d80g714.

Participants were recruited from Gynaecology clinics at Nottingham University Hospitals NHS Trust based on three cohorts as follows: (i) if they were undergoing treatment for endometrioid adenocarcinoma of the endometrium; (ii) if they were undergoing surgery (hysteroscopy, laparoscopy, or laparotomy) for a benign reason, or (iii) if they were attending gynaecological outpatient clinics for investigation and management of PCOS. Patients included in the cancer group had endometroid adenocarcinoma of endometrium confirmed by a previous biopsy and gold-standard histopathological analysis. Participants with PCOS were diagnosed using the Rotterdam criteria (2004) [48]. Other causes of oligo or anovulation were excluded from this study (e.g. congenital adrenal hyperplasia, thyroid disorder or pituitary causes). Patients on hormonal treatment, patients who had previously undergone chemotherapy or radiotherapy and patients with non-endometrioid histology of EC were also excluded. The following demographic details were recorded and analysed as potential confounding factors: age, body mass index (BMI) and blood pressure. The concept map of this study is presented in Fig. 1.

### Sample preparation

Peripheral venous blood samples were collected from each patient in 2014 using standard ethylenediaminetetraacetic acid (EDTA). Whole blood was centrifuged at 1000 rcf (relative centrifugal force) for 10 min, in ambient conditions, to separate cells from the plasma; the plasma was stored at −80 °C until spectral analysis in 2020–2024. Immediately prior to spectral analysis, the frozen blood plasma samples were thawed for 30 min at room temperature. For each patient sample, a 100 μL plasma droplet was pipetted onto an aluminium tape (RS Advance Tapes AT502) covered, borosilicate glass microscope slide (Surgipath® X-tra®, Leica), for immediate ATR-FtIR ‘wet’ analysis and 100 μL droplet was pipetted onto a fused silica coverslip ready for immediate Raman ‘wet’ analysis. A further 100 μL blood plasma aliquot was deposited on an aluminium tape-covered borosilicate glass microscope slide for the dry ATR-FtIR analysis. The samples for dry analysis were smeared over a 15 mm^2^ area and allowed to air-dry at room temperature for a minimum of 24 h, under dust cover, prior to spectral measurement.

### Attenuated total reflection Fourier-transform infrared (ATR-FtIR) spectral acquisition

Infrared spectra were obtained from blood plasma samples using a Bruker Tensor 27 FtIR spectrometer equipped with a Helios ATR diamond crystal attachment, of 250 µm diameter aperture, which was pressed onto the sample to be analysed (Bruker UK Ltd, Coventry), operated *via* OPUS (version 5.5) software. The Helios was prior sealed with aluminium tape (RS Advance Tapes AT502) to minimise ambient air ingress and was supplied with purified air from which CO_2_ and water had been excluded (Parker filtration).

#### Wet blood plasma samples

Two spectral acquisitions were recorded *per* wet blood plasma sample, accumulating 360 scans in 5 min *per* each acquisition, with 4 cm^−1^ spectral resolution, 4 mm internal aperture and 10 kHz mirror step velocity. The ATR diamond crystal was rinsed with de-ionised water and cleaned with 99% isopropyl alcohol (Fisher Chemical™) between patient samples. A background spectrum, without any sample in place, was taken prior to each new sample analysis.

#### Dry blood plasma samples

The dry blood plasma measurements were collected from the periphery of the samples to account for the ‘coffee-ring’ effect [49] that occurred due to capillary flow, a common phenomenon when liquid droplets are dried on a surface. Spectra were acquired from 20 different locations, with a 4 cm^−1^ resolution, aperture of 4 mm and 10 kHz mirror step velocity, accumulating 144 scans in 2 min *per* each measured point. The ATR diamond crystal was cleaned between patient samples with de-ionised water, followed by isopropyl alcohol. A background spectrum was taken prior to each new patient sample analysis and at every 10 min interval.

### Raman spectroscopy spectral acquisition

The Raman instrument consisted of an inverted optical microscope (Nikon Eclipse-Ti) equipped with an automated sample stage (H107 with Proscan II controller, Prior Scientific) and a Raman spectroscopy module. For Raman spectroscopy, a 785 nm laser (Toptica, XTRA) was focused with a 60x/0.9 NA (numerical aperture) oil immersion objective. Laser power at the sample was 120 mW. Back-scattered light from the sample was focused onto a silica glass optical fibre connected to a spectrometer (77200, Oriel, Newport, with a 1000 lines/mm ruled diffraction grating) equipped with a cooled back-illuminated deep depletion charge-coupled device (CCD) (DU401A Andor Technology). For each blood plasma sample, Raman spectra were collected as 0.5 × 0.5 mm^2^ raster scans with a 50 μm x-y resolution. Each point spectrum was acquired with a 3 s exposure time, and all spectra acquired from within the same raster scan were averaged into a single, high signal-to-noise ratio Raman spectrum. Two high signal-to-noise ratio Raman spectra were recorded for each specimen, each spectral acquisition lasting 5.20 min.

### Computational analysis

#### Pre-processing methods

The pre-processing of ATR-FtIR spectral data for both ‘wet’ and dry blood plasma samples, as well as of the Raman spectral data for ‘wet’ blood plasma samples, was performed using Quasar open-source data analysis software, a variant of Orange data visualisation and machine learning platform [50]. The ATR-FtIR ‘wet’ and dry raw spectra were firstly truncated to include only the wavenumber range 1800–900 cm^−1^, known as the ‘bio-fingerprint region’. Atmospheric gas correction was performed in Quasar to minimise any adventitious water vapour and CO_2_ atmospheric spectral artefacts still present. The spectra were baseline corrected using an asymmetric least squares method (smoothing constant of 1000, 10 maximum iterations and 0.05 weighting deviations). Vector normalisation was subsequently applied. The Raman spectra were wavenumber-calibrated using the bands from a polystyrene sample prior to each measurement. Raman spectra were truncated to the wavenumber range of 1800–750 cm^−1^, and a rubber band baseline correction was applied. Normalisation was performed by scaling the Raman spectra to ensure the CH_2_ band at 1450 cm^−1^ was kept constant across the dataset (integration between 1378.1 cm^−1^ and 1490.1 cm^−1^). The ATR-FtIR and Raman spectra were pre-processed separately and then merged using a custom Python code within the Quasar environment to generate a combined dataset for classification analysis. The methods for pre-processing of the combined ATR-FtIR/Raman spectra followed the steps described above in this Section, except for the normalisation procedure, which was as follows. In the combined model, vector normalisation was applied to both the ATR-FtIR and the Raman spectra pre-concatenation, to correct for differences in absorbance intensities across spectra and allow merging and direct comparison between the two techniques.

#### Unsupervised PCA analysis and supervised classification methods

Each spectroscopic technique’s spectra were analysed using PCA for unsupervised feature identification and supervised classification.

In the unsupervised analysis, PCA was applied to the pre-processed ATR-FtIR and Raman spectra of cancer patients and controls, without predefined classes or target labels (cancer or control), to identify spectral patterns and clustering. This approach facilitated better understanding of intrinsic variance in the datasets. Readers are referred to [51] for a comprehensive description of PCA.

In the supervised classification, patients were randomly allocated to a training group (70%) and a testing group (30%). The training dataset was used for model construction, while the testing dataset for model evaluation, ensuring classifiers were evaluated on spectra from patients not seen during training, as in [27]. This randomisation was repeated ten times, generating ten independent training/testing iterations, to assess the impact of patient sampling on classifiers' performance and reproducibility of the results. After training/testing groups were created, class labels (cancer or control) were assigned according to the gold-standard histopathological analysis and spectral pre-processing was conducted. PCA was applied to reduce the dimensionality of all the ATR-FtIR and Raman pre-processed spectral data, while retaining 95% variance. To prevent overfitting, classifiers were trained on the PCA-reduced dataset with 10-fold cross-validation. Four supervised classifiers were explored: Random Forest (RF), Logistic Regression (LR), Support Vector Machine (SVM) and *k*-Nearest Neighbors (*k*NN) algorithm. For methods’ details, we refer the reader to [52, 53], noting that the built-in classifiers from Quasar were used in this study. The trained models were subsequently tested on the residual unseen 30% patient dataset in each iteration.

#### Selection of spectral biomarkers

Following the supervised classification, the ‘Rank’ scoring function in Quasar was used to select a panel of ten classifying wavenumbers. The ‘Rank’ function applies selected machine learning methods (e.g. Random Forest or Logistic Regression) to score wavenumbers, according to their correlation with a target class [54]. Therefore, the wavenumbers with the highest score best correlate with the cancer class, allowing classification between cancers and controls. Among the machine learning methods explored in this paper, Random Forest was alone used with the ‘Rank’ function in Quasar to select a panel of ten wavenumbers, because its overall classification accuracy was superior to that of Logistic Regression and comparable to the overall accuracy of SVM and *k*NN classifiers, across all spectroscopic analyses.

### Statistical analysis

Statistical analysis was performed using Quasar (version 1.9.0) and MATLAB (version R2023a). Validation of spectral consistency of the PCOS and healthy participants’ groups, prior to combining them in a single control group, was performed by multivariate analysis of variance (MANOVA) test. The MANOVA tests were applied to the pre-processed and PCA-transformed spectra of the PCOS and healthy participants’ groups in the ATR-FtIR and Raman analyses. Sensitivity, specificity and accuracy were calculated for each of the ten independent training/testing iterations for all classifiers considered. The mean, standard deviation (SD), confidence intervals (CI), and coefficients of variation for each metric were generated. The classifiers’ performance was analysed and visualised with receiver operating characteristic (ROC) curves and confusion matrices. The area under the curve (AUC) values were calculated. Significant *p* values were subsequently obtained through analysis of variance (ANOVA) tests, applied to each wavenumber in the classifying panels. Clinical differences between cancer and control groups were considered, and *p* values calculated using a *t*-test for age comparison and a Pearson’s chi-squared test of independence for BMI and blood pressure, as in [28]. Statistical significance was determined using a *p* value threshold of 0.05 for all analyses.

## Results

### Participant Demographics

Patients’ characteristics are summarised in Table 1. The EC group was significantly older than the control group (mean age: 63 years compared to 39 years, *p* < 0.0001). Within the control group, 18 patients with PCOS had a mean age of 32 years (SD = 6) and 14 healthy participants had a mean age of 48 years (SD = 15). The cancer group had higher blood pressure levels than the control group (*p* = 0.020), conversely, there was no statistically significant BMI difference between cancers and controls (*p* = 0.274). Disease stage was reported according to the International Federation of Gynaecology and Obstetrics (FIGO) 2009 system [55] as follows: 5 patients had stage IA EC cancer, 7 patients had stage IB, 6 patients had stage IIA, 1 patient had stage IIB, 1 patient had stage IIIA and 2 patients had stage IIIB; there were no patients with stage IV.

**Table 1:** Patient characteristics.

Patient characteristics	Cancers (*n* = 22)	Controls (*n* = 32)	All (*n* = 54)
Age in years			*p* < 0.0001
Mean (SD)	63 (10)	39 (13)	49 (17)
>50	20	6	26
<50	2	26	28
Weight (BMI)			*p* = 0.274
Underweight (<18)	0	0	0
Normal weight (18.5–24.9)	1	1	2
Overweight (25–29.9)	8	17	25
Obese (30–39.9)	11	14	25
Severely obese (>40)	2	0	2
Blood pressure			*p* = 0.020
Normotension	6	19	25
Hypertension^a^	16	13	29

*SD*: standard deviation, *p*: *p* value.

^a^Defined as clinic reading ≥140/90 mmHg [49].

### Unsupervised exploratory analysis

The comparison of spectra from PCOS individuals with spectra from healthy participants showed no statistically significant differences, based on MANOVA tests. These findings confirm the consistency of the dataset within the control group.

The MANOVA tests’ statistical output, score plots and pre-processed mean spectra of PCOS and healthy individuals are presented in Fig. S1 and S2, in the Supplementary Materials section.

**Fig. 2:**
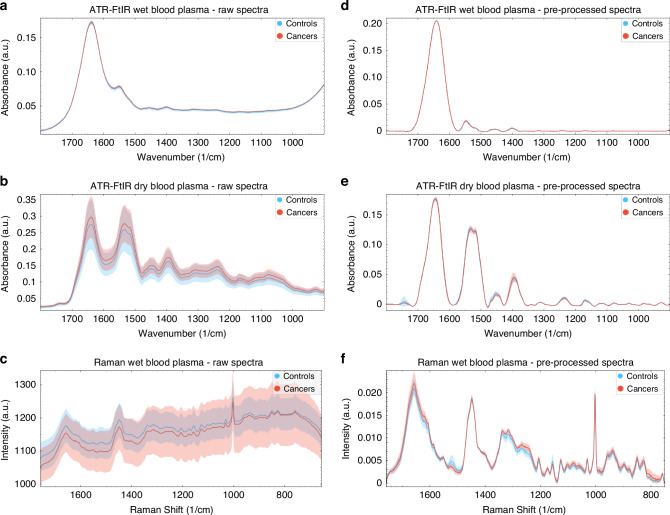
Raw and pre-processed spectra of cancer and control groups. Raw spectra of **a** ATR-FtIR of ‘wet’ blood plasma, **b** ATR-FtIR of dry blood plasma and **c** Raman of ‘wet’ blood plasma. Pre-processed spectra of **d** ATR-FtIR of ‘wet’ blood plasma, **e** ATR-FtIR of dry blood plasma, **f** Raman of ‘wet’ blood plasma. Pre-processing consisted of spectra truncation to the wavenumber range of 1800–900 cm^−1^ for ATR-FtIR and 1800–750 cm^−1^ for Raman, followed by asymmetric least squares baseline correction and vector normalisation. The average spectra are depicted as a turquoise (controls) and red (cancers) lines, and the filled regions denote the variation between datasets.

**Fig. 3:**
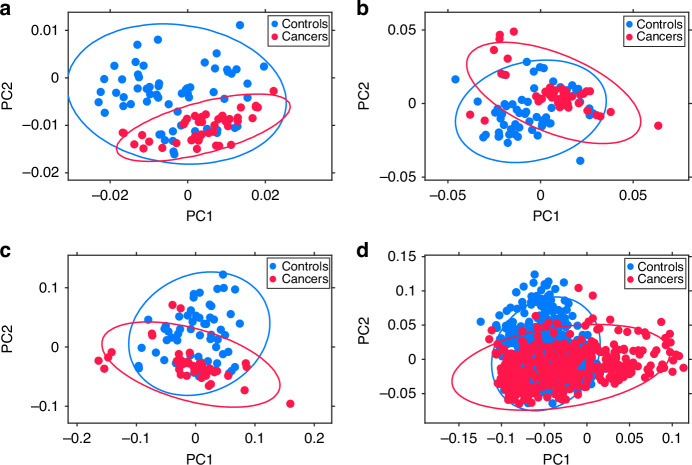
Unsupervised Principal Component Analysis (PCA) scatter plots. Unsupervised Principal Component Analysis (PCA) scatter plots of **a** ATR-FtIR of ‘wet’ plasma, **b** Raman of wet plasma, **c** merged ATR-FtIR and Raman of wet plasma and **d** ATR-FtIR of dry plasma. Unsupervised PCA was applied to explore the intrinsic variance in the data and the relationship between EC and controls spectra. Note that the control group includes healthy participants and patients with PCOS. The principal components (PC) explaining 95% of the dataset variance were identified and PC1 and PC2 are shown. The blue and red data points represent control and cancer groups, respectively. The ellipses represent the 95% confidence intervals (CI) for each group.

**Fig. 4:**
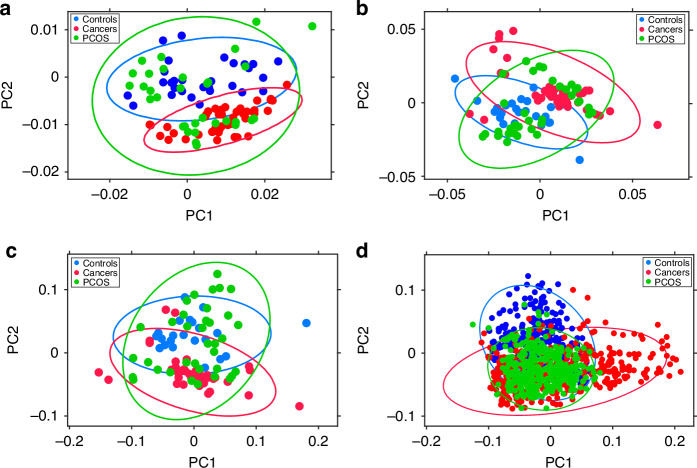
Unsupervised Principal Component Analysis (PCA) scatter plots with PCOS sub-analysis. Unsupervised PCA was applied to the same dataset as in Fig. 2: **a** ATR-FtIR of ‘wet’ plasma, **b** Raman of ‘wet’ plasma, **c** merged ATR-FtIR and Raman of ‘wet’ plasma and **d** ATR-FtIR of dry plasma. Note that here the spectra of patients with PCOS are visualised separately (in green) from those of healthy controls. The principal components (PC) explaining 95% of the dataset variance were identified. PC1 and PC2 are shown. The blue data-points represent the controls (healthy participants), the green data-points represent participants in the PCOS group and the red data-points represent patients in the cancer group. The ellipses represent the 95% confidence intervals (CI) for each group.

The unsupervised analysis with PCA was then conducted on the pre-processed ATR-FtIR and Raman spectra of ‘wet’ and dry blood plasma samples from control and cancer participants. Figure 2 depicts the raw and pre-processed spectra for both ATR-FtIR and Raman, control and cancer datasets. The unsupervised analysis was also used to further explore the characteristics of PCOS spectra within the control group (Figs. 3 and  4).

While the scatter plots in Fig. 3 reveal some overlap between blue (control) and red (cancer) data points, distinct patterns of separation between cancer and control clusters are present. Spectral clustering is also evident in Fig. 4. Here, the control group dataset is further split into healthy participants (in blue) and patients with PCOS (in green), to observe the relationship between PCOS spectra and cancer/control spectra. The overlap of green and red data points highlights spectral similarities between the PCOS and the cancer groups in all scatter plots.

### Supervised analysis of independent and combined ATR-FtIR and Raman for EC diagnosis

#### ‘Wet’ blood plasma analysis using independent and combined ATR-FtIR and Raman spectroscopies

**Fig. 5:**
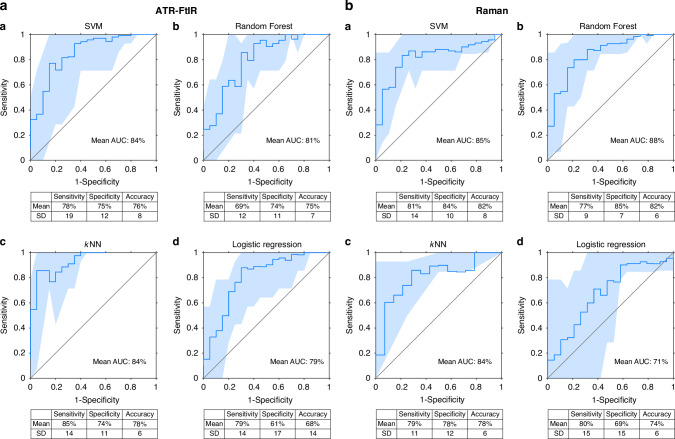
Analyses of classifiers performance for EC detection. **a** ATR-FtIR and **b** Raman spectroscopies of ‘wet’ blood plasma: analyses of classifiers performance for EC detection. The mean AUC of the calculated ROC curves for the ten training/testing iterations are shown, together with sensitivity, specificity and overall accuracy. The following classification systems were applied to the pre-processed spectra after PCA: A) SVM, B) RF, C) *k*NN and D) LR. Note that the mean values (bold turquoise lines) and the standard deviations (turquoise areas) are displayed. AUC area under curve, ROC receiver operating characteristic, SD standard deviation, SVM support vector machine, RF random forest, *k*NN *k-*nearest neighbors and LR logistic regression.

**Fig. 6:**
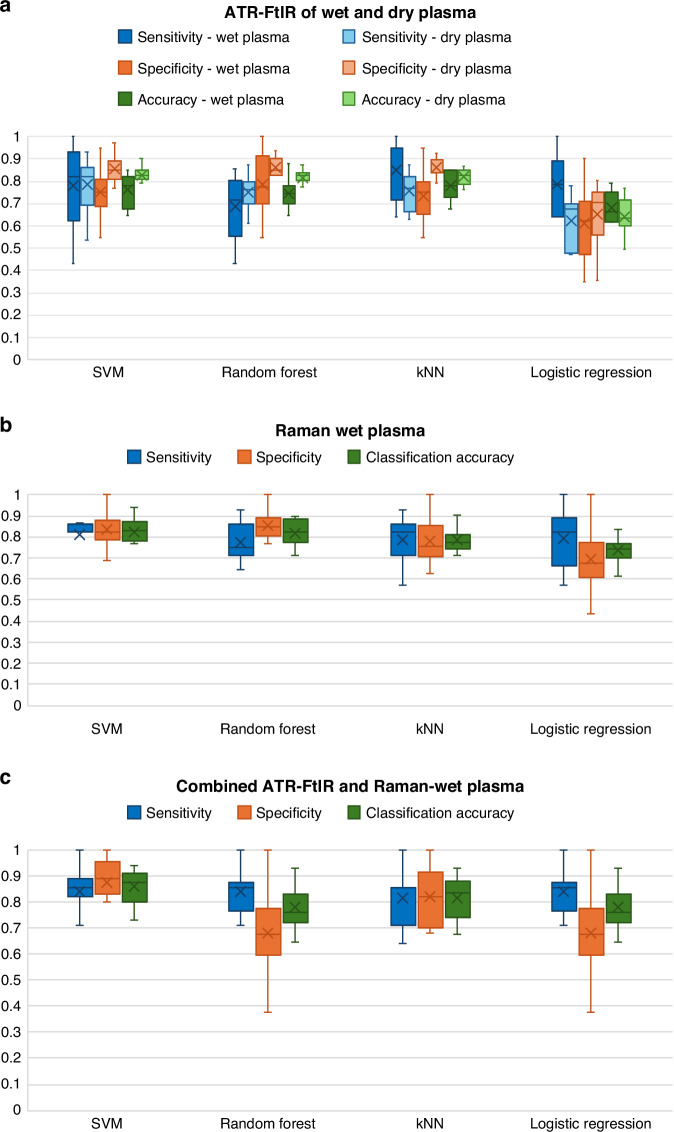
Performance range of classification models across the ten training/testing iterations. **a** ATR-FtIR analysis of ‘wet’ and dry blood plasma; **b** Raman and **c** combined ATR-FtIR-Raman analysis of ‘wet’ plasma. Sensitivities, specificities and accuracies are depicted of for each of SVM, Random Forest, *k*NN and Logistic Regression classifications. The box plots show the performance range of the four classification models across the ten training/testing combinations. The crosses represent the mean values. The whiskers show the spread of sensitivity, specificity and accuracy values among the ten training/testing iterations. The narrower the quartiles, the lesser the values variation. SVM support vector machine, *k*NN *k*-nearest neighbors.

The EC diagnostic abilities of ATR-FtIR and Raman Spectroscopy of ‘wet’ blood plasma were explored with the supervised classification analysis in two ways: first, independently and then in combination. A comprehensive account of test performance for each of the ten training/testing iterations is provided in Supplementary Tables 1–4. Here we report the averaged values of the ten training/testing iterations for each statistic. Figures 5, 6a, b summarise the diagnostic performance of independent ATR-FtIR and Raman analysis of ‘wet’ blood plasma. The highest diagnostic performance from ‘wet’ samples was achieved with the *k*NN classifier for the ATR-FtIR analysis (85% sensitivity, 74% specificity, 78% accuracy) and with the SVM classifier for the Raman analysis (81% sensitivity, 84% specificity, 82% accuracy). The ability to diagnose EC by combining both the ATR-FtIR and Raman ‘wet’ blood plasma spectral data together, using spectra ‘concatenation’, is reported in Fig. 6c. SVM applied to the concatenated spectral analysis was the best-performing classifier, detecting EC with 84% sensitivity, 88% specificity and 86% overall accuracy.

#### Comparison of ‘wet’ and dry blood plasma analysis using ATR-FtIR spectroscopy for EC diagnosis

Figure 6a presents sensitivity, specificity, and accuracy ranges for both dry and ‘wet’ blood plasma samples analy*s*ed using ATR-FtIR spectroscopy. Detailed results for the ‘wet’ samples are reported in Fig. 5. For dry blood plasma, ATR-FtIR achieved sensitivities of 79% (SVM, SD = 12), 75% (RF, SD = 7), 76% (*k*NN, SD = 8), and 62% (LR, SD = 11). Specificities were 86% for SVM, RF, and *k*NN (SD = 6, 4, and 5, respectively), and 66% for LR (SD = 13). AUC values were 90% (SVM, SD = 7), 89% (RF, SD = 4), 88% (*k*NN, SD = 4), and 71% (LR, SD = 10). Overall accuracies were 83% (SVM, SD = 5), 82% (RF, SD = 4), 82% (*k*NN, SD = 3), and 64% (LR, SD = 8).

#### Selection of spectral biomarkers

**Fig. 7:**
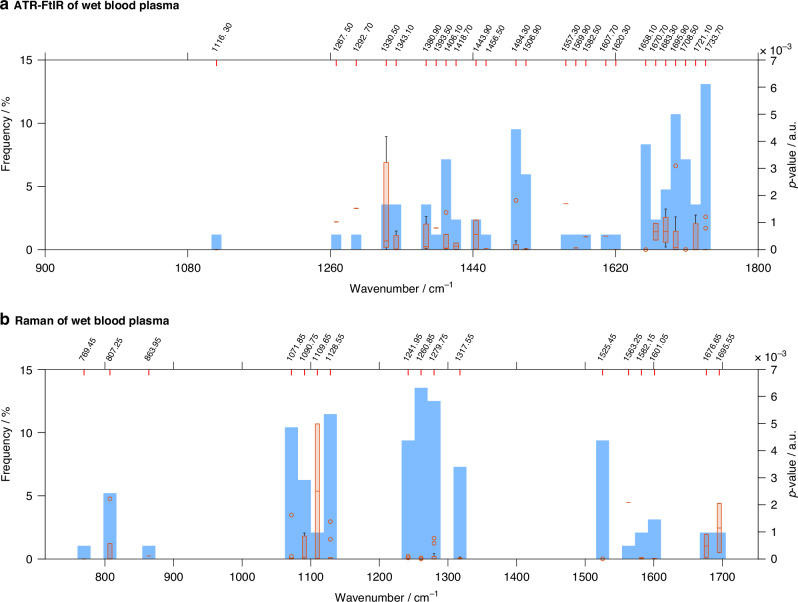

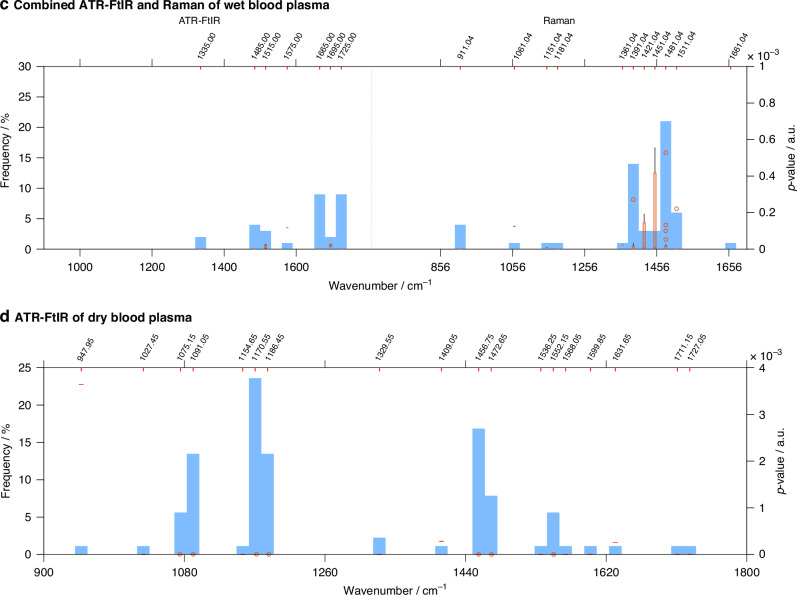
ANOVA analysis applied to the panels of classifying wavenumbers, selected by Rank-Random Forest, after spectra pre-processing and PCA. **a** ATR-FtIR spectra, **b** Raman spectra and **c** combined ATR-FtIR-Raman spectra of ‘wet’ blood plasma; **d** ATR-FtIR spectra of dry blood plasma. Only the statistically significant wavenumbers (*p* < 0.05) from the classifying panels are shown. The frequency of occurrences of these classifying wavenumbers among the ten training/testing iterations is displayed as blue bars (bins); the width of each bin is set at 12.6 cm^−1^, this being a compromise between resolution and signal noise. The orange highlighted intercepts of wavenumbers along the upper *x*-axis are the centre value of each blue bin. The orange box plots within each blue bin represent the range of *p* values for the wavenumbers included in each bin. When the range of *p* values in each bin is very narrow, only the mean values are displayed (orange horizontal lines). For very low *p* values (*p* < 0.0001), the box plots are not visible in the graph, and the blue bins appear empty. The whiskers (in black) identify the highest and the lowest *p* values of the wavenumbers for each region of interest. The circles represent *p* values outliers.

Further analysis of the spectral data from both ATR-FtIR and Raman techniques was conducted to identify a panel of classifying spectral biomarkers. Although the classifying wavenumbers varied across the ten training/testing iterations, recurring peaks enabled the identification of spectral ‘regions of interest’. Statistically significant spectral ‘regions’, along with their recurring wavenumbers and *p* values, are presented in Fig. 7. The centre wavenumbers for these ‘regions’ in both ATR-FtIR and Raman spectroscopies of ‘wet’ and dry blood plasma are summarised in Table 2.

**Table 2:** Spectral regions of interest identified by Rank-Random Forest.

A	ATR-FtIR wet blood plasma	
	1733 cm^−1^	C-O stretching of lipid region
	1708, 1695, 1683, 1670, 1658 cm^−1^	β-sheet, α-helix and random-coil of proteins, Amide I region)
	1506, 1494 cm^−1^	C-H bending vibration from the phenyl rings, Amide II region
	1456, 1443 cm^−1^	CH_3_ bending vibrations of proteins and lipids
	1406, 1380 cm^−1^	CH_3_ bending modes of the methyl groups of proteins
	1343, 1330 cm^−1^	CH_2_ wagging of proteins, Amide III region

	ATR-FtIR dry blood plasma	
	1552 cm^−1^	N‒H bending and C‒N stretching of Amide II region, proteins
	1472 cm^−1^	CH_2_ vibrational modes of methyl and methylene groups of lipids
	1456 cm^−^^1^	CH_3_ bending vibrations of proteins and lipids
	1186, 1170 cm^−1^	C‒O stretching mode of C‒OH groups of serine, threonine and tyrosine of proteins
	1091, 1075 cm^−1^	symmetric PO_2_^−^ stretching in RNA and DNA

	Raman wet blood plasma	
	1525 cm^−1^	C-C stretch modes of carotenoids
	1317 cm^−1^	CH_3_CH_2_ wagging mode present in collagen and purine bases of nucleic acids
	1279 cm^−1^	phosphodiester groups in nucleic acids
	1260, 1241 cm^−1^	Amide III of collagen
	1128 cm^−1^	C-O and C-N stretching vibrations of proteins
	1090 cm^−1^	C‒N stretching mode of proteins and PO_2_ stretching vibration of nucleic acids
	1071 cm^−1^	C‒C or C‒O stretching mode of phospholipids
	807 cm^−1^	PO_2_ stretching vibrations of nucleic acids

B	Combined ATR-FtIR and Raman of wet blood plasma			
	ATR-FtIR	Tentative peak assignment	Raman	Tentative peak assignment
	1725 cm^−1^	ester carbonyl of lipid region	1511 cm^−1^	Amide II region, C-C stretch modes of carotenoids
	1665 cm^−1^	α-helix and random-coil of proteins, Amide I region	1481 cm^−1^	C-C stretching vibrations of adenine and guanine and C-H deformation of lipids
	1515 cm^−1^	C-H bending vibrations of proteins in Amide II region	1391 cm^−1^	C-H rocking of nucleic acids
	1485 cm^−1^	CH2 bending of the methylene chains in lipids	1061 cm^−1^	PO2 stretching from nucleic acids
	1335 cm^−1^	Amide III region, collagen	911 cm^−1^	C-O-C stretching from carbohydrates

The centre wavenumbers of each region are reported for: (A) ATR-FtIR analysis of ‘wet’ blood plasma, Raman analysis of ‘wet’ blood plasma, ATR-FtIR analysis of dry blood plasma and (B) combined ATR-FtIR-Raman analysis of ‘wet’ blood plasma [50–53].

For the ATR-FtIR dataset, the predominant markers for EC were found in the lipid and in the protein regions. Conversely, Raman spectral markers of EC were mainly contributed by the nucleic acids’ bands and by proteins’ and collagen’s bands. Similar results were obtained in the combined ATR-FtIR-Raman analysis, where the absorption peaks appeared consistent with those identified in the independent analyses: the spectra biomarkers contributed mainly by ATR-FtIR are in the lipids’ and proteins’ regions (1725, 1485 cm^−1^ and 1665, 1515, 1335 cm^−1^, respectively), while the spectral markers contributed by Raman are mainly in the nucleic acids’ (1481, 1391 and 1061 cm^−1^), carbohydrates’ (911 cm^−1^) and Amide II (1511 cm^−1^) regions.

## Discussion

This study primarily aimed to investigate the EC detection abilities of ATR-FtIR and Raman spectroscopies using ‘wet’ and dry blood plasma.

The independent Raman analysis of ‘wet’ blood plasma with the SVM classifier achieved 82% overall accuracy, displaying higher detection rates for EC compared to the ‘wet’ blood plasma analysis with ATR-FtIR (78% overall accuracy with *k*NN). The ‘wet’ Raman analysis results were comparable to the dry blood plasma analysis using ATR-FtIR, which detected EC with 83% overall accuracy (SVM classifier). Notably, the combined ATR-FtIR-Raman approach exhibited the highest classification accuracy at 86% for ‘wet’ samples, outperforming the individual techniques.

The identification of spectral biomarkers for EC using ATR-FtIR and Raman spectroscopies highlights the distinct contributions of each technique. ATR-FtIR primarily revealed spectral biomarkers for EC in the lipids’ bands (at 1733 cm^−1^ and 1443 cm^−1^) and protein regions (Amide I, Amide II and Amide III). Conversely, the spectral bands selected by Raman spectroscopy originated mainly from the nucleic acids (at 1317, 1279, 1090, 1071 cm^−1^) and from proteins and collagen’s bands in the Amide III region (1260, 1241 cm^−1^). The complementary nature of these methods becomes apparent in the combined analysis. Here, almost mutually exclusive bands are seen, which are also consistent with the absorption peaks identified in the independent ATR-FtIR and Raman analyses. Importantly, while ‘wet’ and dry blood plasma samples share some spectral features, the significant differences in specific biomarker regions highlight the importance of the blood plasma sample hydration state in spectral analysis.

The association between the presence of endometrial cancer (EC) and alterations in lipid content, changes in protein secondary structure, and shifts in DNA and RNA stretching vibrations has been previously reported [25–28, 32, 56, 57]. These changes likely reflect fundamental biochemical alterations associated with the altered metabolic and proliferative states of cancer cells in tumorigenesis. Dysregulated lipid metabolism, including increased lipid uptake, lipogenesis, and lipid turnover, has been observed in cancer cells and may contribute to tumour growth and invasiveness [58–60]. Additionally, the protein dysregulation of cancer cells, including those involved in cellular signalling and structural integrity, may explain the spectral differences in protein bands between cancer and control groups [61, 62]. Finally, cancer cells exhibit high proliferation rates and increased nuclear-to-cytoplasmic ratios, as elevated cell division rates lead to upregulated nucleic acid synthesis and turnover [63–66]. This enhanced nucleic acid activity, crucial for rapid cell division, could explain the observed spectral changes in the nucleic acid regions. Taken together, these findings highlight how ATR-FtIR and Raman spectroscopies could provide insights into the complex biochemical alterations that occur in cancer development.

The superior diagnostic performance observed with Raman spectroscopy of ‘wet’ blood plasma compared to ATR-FTIR be attributed to the distinct principles underlying each technique. Functional groups with non-polar bonds and large, diffuse electron clouds will scatter light more easily and will be Raman active. Conversely, molecules with functional groups that have strong polar bonds and dipoles display stronger peaks in the IR [67]. Non-atmospheric water is a weak Raman scatterer, thus minimally interferes with Raman signals [68], whereas it causes absorption peaks in ATR-FtIR within the Amide I, Amide II and adjacent band regions. We suspect these peaks may be causing the lower classification performance for ‘wet’ ATR-FtIR spectral analyses compared to that of ATR-FtIR of dry samples and Raman of ‘wet’ samples.

Combining the spectral datasets from both techniques likely mitigated hydration-related challenges, enhancing the synergy of sample characterisation, and leading to improved disease detection rates compared to either method alone (Table 2).

Of note, the undesirable variability in sensitivity, specificity, and accuracy among the ten training/testing iterations was higher for the ‘wet’ samples using both techniques, indicating lower reproducibility compared to dry ATR-FtIR analyses. The acquisition of raw spectral data of ‘wet’ blood plasma samples, for each of the ATR-FtIR and Raman spectrometers, took ~11 min per patient sample, during which the ‘wet’ blood plasma samples may have undergone slight dehydration. This may have contributed to introducing sample-to-sample variability, likely manifesting as spreading of the statistics in both techniques. To mitigate this issue, shortening the spectral acquisition time may be beneficial. Although reducing spectral resolution and the number of co-added scans could expedite data collection, such changes may also decrease the signal-to-noise ratio. In contrast, the dehydrated patient samples remained stable throughout the measurements.

Due to the perceived challenges imposed by the presence of water in biological samples on spectral acquisition, cancer studies with ATR-FtIR spectroscopy have traditionally uniquely favoured the use of dried blood plasma and serum samples [25–27]. The largest multicentric ATR-FtIR spectroscopy study to date of dry blood plasma, by Paraskevaidi et al. [28], reported 83% overall accuracy for discrimination of EC (across all stages) from controls and 78% diagnostic accuracy for sub-analysis of stage I disease *versus* controls [28]. Our study found that ATR-FtIR spectroscopy of dry blood plasma yielded diagnostic results comparable to those reported by Paraskevaidi et al. in their analysis of controls versus EC (across all stages) [28]. Notably, the diagnostic performance of our ‘wet’ blood plasma analysis using ATR-FtIR was comparable to their sub-analysis of controls *versus* stage I disease. Our cancer group included 50% of patients with stage I disease.

The results here, together with those of Paraskevaidi et al. [28], highlight the promising role of ATR-FtIR spectroscopy as an early diagnostic tool for EC, and, importantly, the feasibility of applying ATR-FtIR to ‘wet’ blood plasma samples for early-stage disease detection. With regard to Raman spectroscopy, only one preliminary study, by Artemyev et al., investigated surface-enhanced Raman scattering (SERS) for EC diagnosis with dry blood plasma samples [69], reporting 66% sensitivity, 95% specificity and 85% accuracy for discrimination of EC *versus* controls. Our Raman analysis of ‘wet’ blood plasma achieved comparable diagnostic accuracy to that reported by Artemyev et al. and superior sensitivity. Moreover, our combined ATR-FtIR-Raman approach achieved the highest EC detection rates overall, demonstrating that the techniques’ synergy can be harnessed to improve diagnostic performance.

Finally, the unsupervised exploratory analysis with PCA of ATR-FtIR and Raman spectra, conducted to evaluate the intrinsic variance of the data, demonstrated spectral clustering between cancers and controls in each of the independent and combined spectroscopy analyses (Fig. 3). Denser clusters reflected higher within-cluster spectral similarities. These were particularly evident in the ATR-FtIR dataset of dry blood plasma. On the other hand, the slightly higher within-clusters data spread, seen for the ATR-FtIR and Raman ‘wet’ analyses, might be a consequence of adventitious sample dehydration potentially occurring during spectral acquisition. Of note, some overlap was present between cancer and control clusters for both ATR-FtIR and Raman analyses. Interestingly, analysing the spectra of patients with PCOS separately from the healthy women in the control group significantly reduced the overlap between cancer and healthy controls. In contrast, the clusters of the PCOS dataset exhibited substantial overlap with both the healthy individuals and cancer groups (Fig. 4), suggesting that PCOS spectra share similarities with both groups. This could indicate that some PCOS individuals exhibit metabolic or molecular profiles that are closer to healthy individuals, while others show shifts that resemble cancerous changes.

This aligns with the known metabolic heterogeneity of PCOS, which carries a 3 to 4-fold increased risk of EC [70]. Leading theories to explain endometrial carcinogenesis in individuals with PCOS propose the following aetiologies: raised oestrogen levels, obesity and anovulatory menstrual cycles/infrequent shedding of the endometrium, as well insulin resistance, most of which have been shown to promote endometrial proliferation [71]. Nevertheless, the exact molecular mechanisms that increase the risk of EC in PCOS remain unclear [72]. This preliminary work on PCOS presents, for the first time, spectral similarities between the blood plasma of patients with EC and PCOS. The observed spectral overlap may reflect early biochemical changes preceding carcinogenesis. Alternatively, the spectral variability within PCOS may simply highlight its biochemical diversity, causing its spectra to be positioned between healthy and cancer groups.

To the best of our knowledge, this is the first study to assess the feasibility of using ‘wet’ blood plasma samples for EC detection through ATR-FtIR or Raman. It is also the first study, innovatively, to integrate spectra from both the Raman and ATR-FtIR techniques, resulting in a truly combined analysis.

Strengths of the study include the accompanying rigorous participant assessment *via* pipelle biopsy, ensuring accurate allocation to cancer and control groups based on histopathology. Additionally, our computational analysis was designed to be numerically robust and to evaluate diagnostic classification reproducibility. Thus, we applied the classification algorithms to ten randomly generated training/testing iterations of patient datasets. This enabled generation of ten independent training, validation and testing models, to verify precision of the classifiers’ performance. We utilised aluminium tape-covered substrates for ATR-FtIR analysis, which exhibit minimal infrared absorption, while enabling collection of key biochemical features [56, 73]. This approach is cost-effective, accessible, one-use yet sustainable (aluminium foil is sterilised during remelting for recycling; glass microscope slide substrates are sterilised and reused in-house), achieving organic-cleansed, recycled option for clinical settings, compared to expensive alternatives like calcium fluoride (CaF_2_) slides. In all, aluminium tape-covered substrates for ATR-FtIR analysis are suitable for adoption in wide-scale testing strategies and in low-income settings in EC cancer screening, and beyond.

The study has limitations. The small sample size of 54 participants, while consistent with feasibility study recommendations [45–47, 74], may limit generalisability. Additionally, there were differences in age and hypertension rates between cancer and control groups, primarily due to the inclusion of younger participants with PCOS in the control group. While a previous larger multicentric study suggested that age, BMI, and blood pressure did not significantly influence ATR-FtIR spectral classification of dried blood plasma for EC, indicating that the technique’s diagnostic capabilities were related to disease presence/absence [28], future studies applying ATR-FtIR and Raman to ‘wet’ blood analyses should continue accounting for these confounding factors. Our study only included patients with endometrioid adenocarcinoma of the endometrium. It is important to note that some subtypes of endometrial cancer, particularly endometrioid, may have a secretory nature, which could indeed make them more amenable to biospectroscopy tests of peripheral blood. As such, the findings of this study will not be applicable to other non-secretory subtypes of EC, which have distinct molecular and/or clinical characteristics and so could present different diagnostic challenges. Finally, our control group included both healthy participants and individuals with PCOS, which may have led to an underestimation of the classifiers’ diagnostic abilities, as the PCOS spectra showed similarities to the EC group in the unsupervised PCA analysis.

## Future research directions

This study has demonstrated the feasibility of using vibrational biospectroscopy techniques to analyse ‘fresh’ (wet) blood samples for endometrial cancer detection, highlighting their potential for rapid and cost-effective blood analysis. Our findings have significant implications for the future development of bedside diagnostic approaches for EC and population-level screening.

However, several key aspects must be addressed before clinical implementation. Our results should be validated in larger, adequately powered, prospective studies. We propose multicentric participation, with collaboration of primary care and secondary care partners to reach high-risk demographics. Coordination could be achieved through regional or national cancer networks. Significantly, the experimental design might involve frozen blood samples from research network partners being sent to a central location for EC analysis, ensuring protocol standardisation and consistency.

Future research should focus on diverse populations, including individuals from ethnic minority backgrounds, and continue to account for EC risk factors in the analysis of potential confounders. Studies should also expand to include a broader range of EC histotypes and explore the diagnostic potential of spectroscopy techniques for pre-cancerous lesions such as endometrial hyperplasia.

Additionally, the role of biospectroscopy techniques as a potential EC diagnostic tool in high-risk individuals with PCOS warrants further investigation; research is needed to enable further spectral characterisation, focusing on exploring potential carcinogenesis biomarkers and assessing biospectroscopy diagnostic performance in this patient group.

These future approaches will help enhance the diagnostic efficiency and generalisability of the applied spectroscopic techniques, supporting the development of both early EC detection and EC screening methods.

## Conclusions

In summary, this study highlights the significant potential of vibrational biospectroscopy techniques in advancing EC diagnosis, particularly using ‘wet’ blood samples. While the initial findings are promising, larger and more diverse studies are needed to validate these techniques and refine their application in clinical settings. Through further investigation and standardisation, biospectroscopy techniques could become a valuable tool for early EC detection and population-wide screening.

## Supplementary information

Figure S1

Figure S2

Table S1, Table S2, Table S3, Table S4

## Data Availability

Data contributing to this manuscript will be made available upon reasonable request to the corresponding authors.

## Code availability

Codes used for this work will be made available upon reasonable request to the corresponding authors.
