# Supplementary material for: Infrared and Raman spectroscopy of blood plasma for rapid endometrial cancer detection
Source: Br J Cancer. 2025 May 18;133(2):194–207. doi: 10.1038/s41416-025-03050-0 (PMC12304263; doi:10.1038/s41416-025-03050-0)
Supplement: Supplementary file 1 — Figure S1 [file 41416_2025_3050_MOESM1_ESM.pdf]

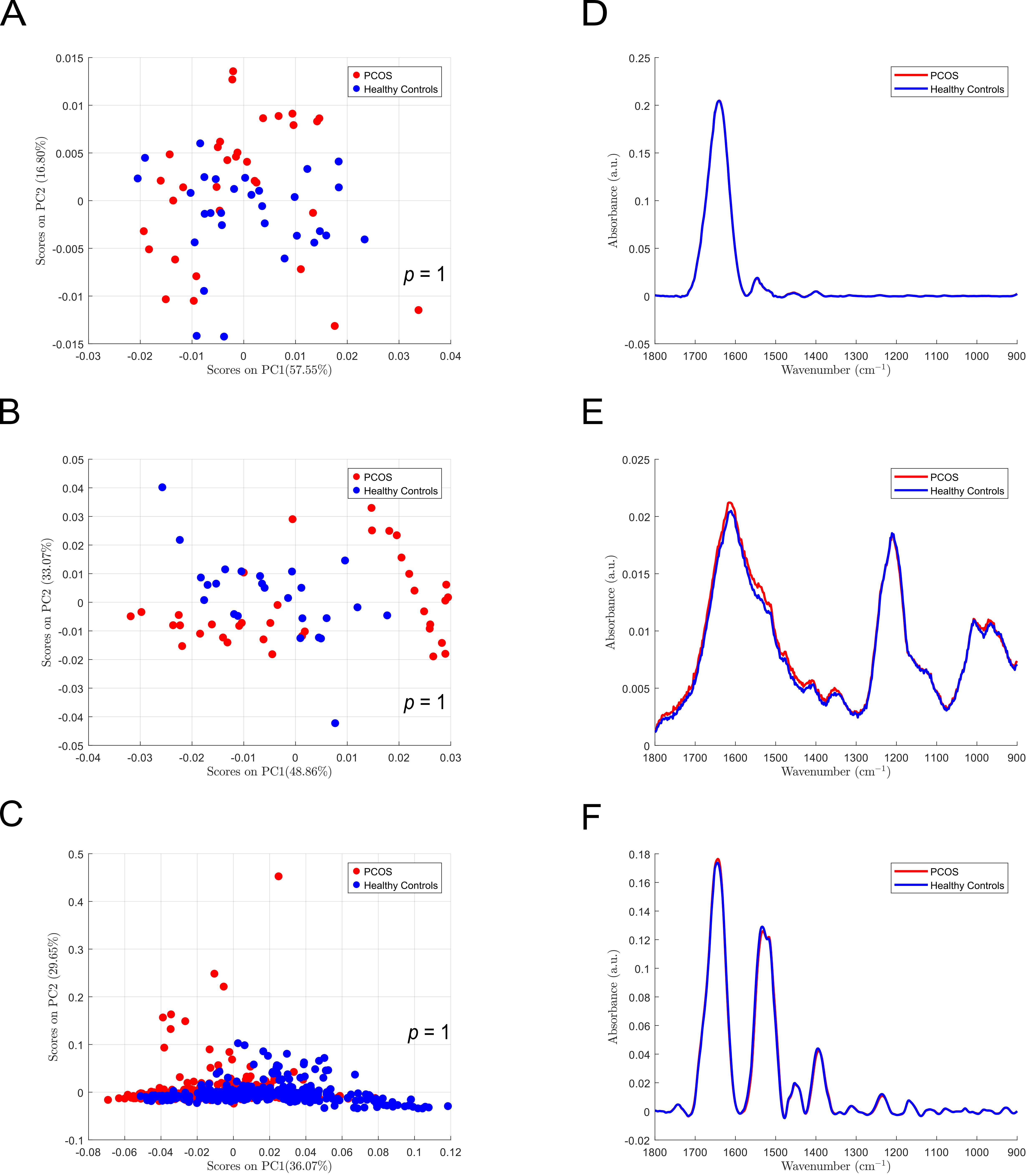

Figure S1. (A, B, C) Score plots generated after unsupervised principal component analysis (PCA) of pre-processed ATR-FtIR and Raman spectra to evaluate consistency between PCOS and healthy individuals. (D, E, F) Pre-processed mean spectra of PCOS and healthy individuals, displayed overlaid for comparison. (A) Score plot and (D) mean spectra for ATR-FtIR spectroscopy of wet blood plasma; (B) score plot and (E) mean spectra for Raman spectroscopy of wet blood plasma; (C) score plot and (F) mean spectra for ATR-FTIR spectroscopy of dry blood plasma. The PCA scores and mean spectra of participants with PCOS are shown in red, while the PCA scores and mean spectra of healthy individuals are shown in blue. The percentage of variance explained by PC1 and PC2 are reported in brackets. P-values were generated using MANOVA tests applied to the pre-processed and PCA-transformed spectral datasets of PCOS and healthy individuals. ATR-FtIR: Attenuated Total Reflection-Fourier Transform Infrared; p: p-value; PCOS: polycystic ovary syndrome; PC: principal component; MANOVA: multivariate analysis of variance.
