# Supplementary material for: Infrared and Raman spectroscopy of blood plasma for rapid endometrial cancer detection
Source: Br J Cancer. 2025 May 18;133(2):194–207. doi: 10.1038/s41416-025-03050-0 (PMC12304263; doi:10.1038/s41416-025-03050-0)
Supplement: Supplementary file 2 — Figure S2 [file 41416_2025_3050_MOESM2_ESM.pdf]

| ATR-FtIR Wet Blood Plasma | Raman Wet Blood Plasma | ATR-FtIR Dry Blood Plasma |
|---|---|---|
| MANOVA p-values: 1 | MANOVA p-values: 1 | MANOVA p-values: 1 |
| Statistics: | Statistics: | Statistics: |
| W: [18×18 double]<br>B: [18×18 double]<br>T: [18×18 double]<br>dfW: 60<br>dfB: 1<br>dfT: 61<br>lambda: 0.2760<br>chisq: 65.6542<br>chisqdf: 18<br>eigenval: [18×1 double]<br>eigenvec: [18×18 double]<br>canon: [62×18 double]<br>mdist: [62×1 double]<br>gmdist: [2×2 double]<br>gnames: {2×1 cell} | W: [7×7 double]<br>B: [7×7 double]<br>T: [7×7 double]<br>dfW: 59<br>dfB: 1<br>dfT: 60<br>lambda: 0.52<br>chisq: 35.6982<br>chisqdf: 7<br>eigenval: [7×1 double]<br>eigenvec: [7×7 double]<br>canon: [61×7 double]<br>mdist: [61×1 double]<br>gmdist: [2×2 double]<br>gnames: {2×1 cell} | W: [7×7 double]<br>B: [7×7 double]<br>T: [7×7 double]<br>dfW: 638<br>dfB: 1<br>dfT: 639<br>lambda: 0.5912<br>chisq: 333.4664<br>chisqdf: 7<br>eigenval: [7×1 double]<br>eigenvec: [7×7 double]<br>canon: [640×7 double]<br>mdist: [640×1 double]<br>gmdist: [2×2 double]<br>gnames: {2×1 cell} |

**Figure S2.** Statistical output of multivariate analysis of variance (MANOVA) tests applied to the pre-processed PCA-transformed spectra of PCOS and healthy individual for the ATR-FtIR and Raman blood plasma analyses. W: within-group sum of squares and cross-product matrix; B: between-group sum of squares and cross-product matrix; T: total sum of squares and cross-product matrix; dfW: degrees of freedom within groups; dfB: degrees of freedom between groups; dfT: total degrees of freedom; lambda: Wilks' lambda; chisq: chi-square statistic; chisqdf: chi-square degrees of freedom; eigenval: eigenvalues; eigenvec: eigenvectors; canon: canonical scores; mdist: mahalanobis distances; gmdist: group mahalanobis distances; gnames: group names; p-values: statistical significance. ATR-FtIR: Attenuated Total Reflection-Fourier Transform Infrared; PCA: principal compo-nent analysis; PCOS: polycystic ovary syndrome.
