# Supplementary material for: Infrared and Raman spectroscopy of blood plasma for rapid endometrial cancer detection
Source: Br J Cancer. 2025 May 18;133(2):194–207. doi: 10.1038/s41416-025-03050-0 (PMC12304263; doi:10.1038/s41416-025-03050-0)
Supplement: Supplementary file 3 — Table S1, Table S2, Table S3, Table S4 [file 41416_2025_3050_MOESM3_ESM.docx]

|  | **Logistic regression** | | | |  | **Random Forest** | | | |  | **SVM** | | | |  | ***k*NN** | | | |
| --- | --- | --- | --- | --- | --- | --- | --- | --- | --- | --- | --- | --- | --- | --- | --- | --- | --- | --- | --- |
|  | **Sens** | **Spec** | **AUC** | **Acc** |  | **Sens** | **Spec** | **AUC** | **Acc** |  | **Sens** | **Spec** | **AUC** | **Acc** |  | **Sens** | **Spec** | **AUC** | **Acc** |
| V1 | 0.79 | 0.55 | 0.78 | 0.65 |  | 0.57 | 0.70 | 0.71 | 0.65 |  | 0.93 | 0.80 | 0.89 | 0.85 |  | 0.93 | 0.65 | 0.80 | 0.77 |
| V2 | 0.64 | 0.60 | 0.66 | 0.62 |  | 0.71 | 0.70 | 0.78 | 0.71 |  | 0.64 | 0.65 | 0.64 | 0.65 |  | 0.71 | 0.65 | 0.83 | 0.68 |
| V3 | 0.79 | 0.50 | 0.71 | 0.62 |  | 0.64 | 0.80 | 0.82 | 0.74 |  | 1.00 | 0.70 | 0.94 | 0.82 |  | 0.93 | 0.75 | 0.96 | 0.82 |
| V4 | 1.00 | 0.40 | 0.83 | 0.65 |  | 0.86 | 0.80 | 0.91 | 0.82 |  | 0.93 | 0.75 | 0.87 | 0.82 |  | 1.00 | 0.75 | 0.91 | 0.85 |
| V5 | 0.64 | 0.70 | 0.79 | 0.68 |  | 0.43 | 1.00 | 0.90 | 0.77 |  | 0.57 | 0.95 | 0.92 | 0.79 |  | 0.71 | 0.95 | 0.96 | 0.85 |
| V6 | 0.71 | 0.65 | 0.71 | 0.68 |  | 0.50 | 0.90 | 0.80 | 0.75 |  | 0.43 | 0.85 | 0.79 | 0.68 |  | 0.64 | 0.75 | 0.83 | 0.71 |
| V7 | 1.00 | 0.35 | 0.82 | 0.62 |  | 0.86 | 0.55 | 0.71 | 0.68 |  | 0.86 | 0.55 | 0.81 | 0.68 |  | 1.00 | 0.55 | 0.79 | 0.74 |
| V8 | 0.64 | 0.90 | 0.91 | 0.79 |  | 0.79 | 0.95 | 0.93 | 0.88 |  | 0.86 | 0.80 | 0.88 | 0.82 |  | 0.71 | 0.80 | 0.88 | 0.77 |
| V9 | 0.79 | 0.70 | 0.86 | 0.74 |  | 0.71 | 0.70 | 0.71 | 0.71 |  | 0.79 | 0.70 | 0.84 | 0.74 |  | 0.93 | 0.70 | 0.96 | 0.79 |
| V10 | 0.86 | 0.75 | 0.84 | 0.79 |  | 0.79 | 0.75 | 0.83 | 0.77 |  | 0.79 | 0.75 | 0.81 | 0.77 |  | 0.93 | 0.80 | 0.91 | 0.85 |
|  |  |  |  |  |  |  |  |  |  |  |  |  |  |  |  |  |  |  |  |
| Mean | 0.79 | 0.61 | 0.79 | 0.68 |  | 0.69 | 0.79 | 0.81 | 0.75 |  | 0.78 | 0.75 | 0.84 | 0.76 |  | 0.85 | 0.74 | 0.88 | 0.78 |
| SD | 0.14 | 0.17 | 0.08 | 0.06 |  | 0.15 | 0.14 | 0.09 | 0.07 |  | 0.19 | 0.12 | 0.09 | 0.08 |  | 0.14 | 0.11 | 0.07 | 0.06 |
| CI | 0.09 | 0.10 | 0.05 | 0.04 |  | 0.09 | 0.09 | 0.05 | 0.05 |  | 0.12 | 0.07 | 0.06 | 0.05 |  | 0.09 | 0.07 | 0.04 | 0.04 |
| CV | 0.18 | 0.28 | 0.10 | 0.09 |  | 0.22 | 0.18 | 0.11 | 0.10 |  | 0.24 | 0.16 | 0.11 | 0.10 |  | 0.17 | 0.15 | 0.08 | 0.08 |

**Table S1:** Diagnostic performance of wet blood plasma analysis using ATR-FtIR spectroscopy. Sensitivity, specificity, Area Under the Receiver Operating Characteristic Curve (AUC) and overall accuracy were calculated for Logistic Regression, Random Forest, Support Vector Machine (SVM) and *k-*nearest neighbours (*k*NN) applied to ten training/testing iterations (versions 1 to 10). The mean, standard deviation (SD), confidence interval (CI) and coefficient of variation (CV) are reported for all statistics. Sens: sensitivity; spec: specificity; acc: accuracy; V1-10: versions 1-10.

|  | **Logistic regression** | | | |  | **Random Forest** | | | |  | | **SVM** | | |  | | **kNN** | | |
| --- | --- | --- | --- | --- | --- | --- | --- | --- | --- | --- | --- | --- | --- | --- | --- | --- | --- | --- | --- |
|  | **Sens** | **Spec** | **AUC** | **Acc** |  | **Sens** | **Spec** | **AUC** | **Acc** |  | **Sens** | **Spec** | **AUC** | **Acc** |  | **Sens** | **Spec** | **AUC** | **Acc** |
| V1 | 0.78 | 0.69 | 0.80 | 0.73 |  | 0.73 | 0.90 | 0.89 | 0.83 |  | 0.80 | 0.98 | 0.95 | 0.90 |  | 0.76 | 0.88 | 0.90 | 0.83 |
| V2 | 0.48 | 0.73 | 0.73 | 0.62 |  | 0.69 | 0.84 | 0.84 | 0.77 |  | 0.54 | 0.86 | 0.73 | 0.73 |  | 0.64 | 0.90 | 0.85 | 0.79 |
| V3 | 0.59 | 0.54 | 0.64 | 0.56 |  | 0.79 | 0.87 | 0.89 | 0.84 |  | 0.66 | 0.93 | 0.92 | 0.82 |  | 0.87 | 0.80 | 0.91 | 0.83 |
| V4 | 0.48 | 0.76 | 0.68 | 0.64 |  | 0.87 | 0.83 | 0.94 | 0.84 |  | 0.93 | 0.85 | 0.97 | 0.88 |  | 0.81 | 0.87 | 0.91 | 0.85 |
| V5 | 0.68 | 0.66 | 0.79 | 0.67 |  | 0.82 | 0.83 | 0.91 | 0.83 |  | 0.89 | 0.77 | 0.92 | 0.82 |  | 0.84 | 0.89 | 0.93 | 0.87 |
| V6 | 0.69 | 0.57 | 0.68 | 0.62 |  | 0.76 | 0.85 | 0.90 | 0.81 |  | 0.84 | 0.85 | 0.94 | 0.84 |  | 0.67 | 0.91 | 0.86 | 0.81 |
| V7 | 0.72 | 0.81 | 0.82 | 0.77 |  | 0.61 | 0.83 | 0.81 | 0.74 |  | 0.71 | 0.86 | 0.87 | 0.79 |  | 0.63 | 0.86 | 0.80 | 0.76 |
| V8 | 0.67 | 0.75 | 0.82 | 0.72 |  | 0.79 | 0.94 | 0.94 | 0.88 |  | 0.85 | 0.82 | 0.91 | 0.83 |  | 0.74 | 0.93 | 0.90 | 0.85 |
| V9 | 0.69 | 0.36 | 0.51 | 0.49 |  | 0.76 | 0.86 | 0.85 | 0.82 |  | 0.85 | 0.79 | 0.89 | 0.82 |  | 0.81 | 0.75 | 0.88 | 0.77 |
| V10 | 0.47 | 0.72 | 0.67 | 0.62 |  | 0.71 | 0.92 | 0.91 | 0.83 |  | 0.80 | 0.88 | 0.93 | 0.84 |  | 0.78 | 0.87 | 0.88 | 0.83 |
|  | | | | | | | | | | | | | | | | | | | |
| Mean | 0.62 | 0.66 | 0.71 | 0.64 |  | 0.75 | 0.86 | 0.89 | 0.82 |  | 0.79 | 0.86 | 0.90 | 0.83 |  | 0.76 | 0.86 | 0.88 | 0.82 |
| SD | 0.11 | 0.13 | 0.10 | 0.08 |  | 0.07 | 0.04 | 0.04 | 0.04 |  | 0.12 | 0.06 | 0.07 | 0.05 |  | 0.08 | 0.05 | 0.04 | 0.03 |
| CI | 0.07 | 0.08 | 0.06 | 0.05 |  | 0.05 | 0.03 | 0.03 | 0.02 |  | 0.07 | 0.04 | 0.04 | 0.03 |  | 0.05 | 0.03 | 0.02 | 0.02 |
| CV | 0.18 | 0.21 | 0.14 | 0.13 |  | 0.10 | 0.05 | 0.05 | 0.05 |  | 0.15 | 0.07 | 0.08 | 0.06 |  | 0.11 | 0.06 | 0.04 | 0.04 |

**Table S2:** Diagnostic performance of dry blood plasma analysis using ATR-FtIR spectroscopy. Sensitivity, specificity, Area Under the Receiver Operating Characteristic Curve (AUC) and overall accuracy were calculated for Logistic Regression, Random Forest, Support Vector Machine (SVM) and *k*-nearest neighbours (*k*NN) applied to ten training/testing iterations (versions 1 to 10). The mean, standard deviation (SD), confidence interval (CI) and coefficient of variation (CV) are reported for all statistics. Sens: sensitivity; spec: specificity; acc: accuracy; V1-10: version 1-10.

**Table S3:** Diagnostic performance of wet blood plasma analysis using Raman spectroscopy. Sensitivity, specificity, Area Under the Receiving Operating Characteristic Curve (AUC) and overall accuracy were calculated for Logistic Regression, Random Forest, Support Vector Machine (SVM) and k-nearest neighbours (*k*NN) applied to ten training/testing iterations (versions 1 to 10). The mean, standard deviation (SD), confidence interval (CI) and coefficient of variation (CV) are reported for all statistics. Sens: sensitivity; spec: specificity; acc: accuracy; V1-10: version 1-10.

|  | **Logistic regression** | | | |  | **Random Forest** | | | |  | **SVM** | | | |  | | **kNN** | | |
| --- | --- | --- | --- | --- | --- | --- | --- | --- | --- | --- | --- | --- | --- | --- | --- | --- | --- | --- | --- |
|  | **Sens** | **Spec** | **AUC** | **Acc** |  | **Sens** | **Spec** | **AUC** | **Acc** |  | **Sens** | **Spec** | **AUC** | **Acc** |  | **Sens** | **Spec** | **AUC** | **Acc** |
| V1 | 0.79 | 0.61 | 0.61 | 0.69 |  | 0.79 | 0.89 | 0.84 | 0.84 |  | 0.86 | 0.83 | 0.86 | 0.84 |  | 0.86 | 0.72 | 0.84 | 0.78 |
| V2 | 1.00 | 0.60 | 0.76 | 0.77 |  | 0.93 | 0.85 | 0.94 | 0.88 |  | 0.93 | 0.80 | 0.95 | 0.85 |  | 0.93 | 0.75 | 0.94 | 0.82 |
| V3 | 0.57 | 0.65 | 0.69 | 0.61 |  | 0.71 | 0.77 | 0.84 | 0.74 |  | 0.43 | 0.82 | 0.74 | 0.65 |  | 0.64 | 0.77 | 0.76 | 0.71 |
| V4 | 0.86 | 0.67 | 0.70 | 0.75 |  | 0.71 | 0.89 | 0.89 | 0.81 |  | 0.86 | 0.78 | 0.85 | 0.81 |  | 0.86 | 0.67 | 0.80 | 0.75 |
| V5 | 0.64 | 1.00 | 0.85 | 0.83 |  | 0.79 | 1.00 | 0.96 | 0.90 |  | 0.86 | 1.00 | 0.90 | 0.93 |  | 0.57 | 1.00 | 0.89 | 0.80 |
| V6 | 0.86 | 0.72 | 0.78 | 0.78 |  | 0.71 | 0.83 | 0.87 | 0.78 |  | 0.86 | 1.00 | 0.88 | 0.94 |  | 0.86 | 0.94 | 0.89 | 0.91 |
| V7 | 1.00 | 0.44 | 0.52 | 0.70 |  | 0.86 | 0.82 | 0.90 | 0.83 |  | 0.86 | 0.69 | 0.87 | 0.77 |  | 0.86 | 0.63 | 0.78 | 0.73 |
| V8 | 0.71 | 0.77 | 0.62 | 0.74 |  | 0.64 | 0.77 | 0.77 | 0.71 |  | 0.86 | 0.82 | 0.85 | 0.84 |  | 0.79 | 0.82 | 0.85 | 0.81 |
| V9 | 0.86 | 0.68 | 0.80 | 0.76 |  | 0.71 | 0.84 | 0.87 | 0.79 |  | 0.71 | 0.84 | 0.77 | 0.79 |  | 0.79 | 0.74 | 0.84 | 0.76 |
| V10 | 0.67 | 0.79 | 0.75 | 0.74 |  | 0.87 | 0.90 | 0.90 | 0.88 |  | 0.87 | 0.79 | 0.89 | 0.82 |  | 0.73 | 0.79 | 0.81 | 0.77 |
|  |  |  |  |  |  |  |  |  |  |  |  |  |  |  |  |  |  |  |  |
| Mean | 0.80 | 0.69 | 0.71 | 0.74 |  | 0.77 | 0.85 | 0.88 | 0.82 |  | 0.81 | 0.84 | 0.85 | 0.82 |  | 0.79 | 0.78 | 0.84 | 0.78 |
| SD | 0.15 | 0.15 | 0.10 | 0.06 |  | 0.09 | 0.07 | 0.05 | 0.06 |  | 0.14 | 0.10 | 0.06 | 0.08 |  | 0.11 | 0.12 | 0.06 | 0.06 |
| CI | 0.09 | 0.09 | 0.06 | 0.04 |  | 0.06 | 0.04 | 0.03 | 0.04 |  | 0.09 | 0.06 | 0.04 | 0.05 |  | 0.07 | 0.07 | 0.04 | 0.03 |
| CV | 0.18 | 0.21 | 0.14 | 0.08 |  | 0.12 | 0.08 | 0.06 | 0.08 |  | 0.18 | 0.11 | 0.07 | 0.10 |  | 0.14 | 0.15 | 0.07 | 0.07 |

**Table S4:** Diagnostic performance of wet blood plasma analysis using combined ATR-FtIR – Raman spectroscopies. Sensitivity, specificity, Area Under the Receiver Operating Characteristic Curve (AUC) and overall accuracy were calculated for Logistic Regression, Random Forest, Support Vector Machine (SVM) and k-nearest neighbours (*k*NN) applied to ten training/testing iterations (versions 1 to 10). The mean, standard deviation (SD), confidence interval (CI) and coefficient of variation (CV) are reported for all statistics. Sens: sensitivity; spec: specificity; acc: accuracy; V1-10: version 1-10.

|  | **Logistic regression** | | | |  | **Random Forest** | | | |  | **SVM** | | | |  | **kNN** | | | |
| --- | --- | --- | --- | --- | --- | --- | --- | --- | --- | --- | --- | --- | --- | --- | --- | --- | --- | --- | --- |
|  | **Sens** | **Spec** | **AUC** | **Acc** |  | **Sens** | **Spec** | **AUC** | **Acc** |  | **Sens** | **Spec** | **AUC** | **Acc** |  | **Sens** | **Spec** | **AUC** | **Acc** |
| V1 | 0.86 | 0.78 | 0.88 | 0.81 |  | 0.79 | 0.89 | 0.87 | 0.84 |  | 0.86 | 0.89 | 0.92 | 0.88 |  | 0.86 | 0.83 | 0.86 | 0.84 |
| V2 | 1.00 | 0.60 | 0.95 | 0.77 |  | 0.86 | 0.90 | 0.91 | 0.88 |  | 1.00 | 0.80 | 0.94 | 0.88 |  | 1.00 | 0.75 | 0.96 | 0.85 |
| V3 | 0.71 | 0.71 | 0.81 | 0.71 |  | 0.43 | 1.00 | 0.81 | 0.74 |  | 0.57 | 1.00 | 0.90 | 0.81 |  | 0.71 | 0.88 | 0.78 | 0.81 |
| V4 | 0.86 | 0.67 | 0.81 | 0.75 |  | 0.86 | 1.00 | 0.91 | 0.94 |  | 0.86 | 0.94 | 0.90 | 0.91 |  | 0.86 | 0.89 | 0.88 | 0.88 |
| V5 | 0.79 | 1.00 | 0.89 | 0.90 |  | 0.29 | 1.00 | 0.99 | 0.67 |  | 0.86 | 1.00 | 0.96 | 0.93 |  | 0.86 | 1.00 | 0.92 | 0.93 |
| V6 | 0.86 | 0.78 | 0.85 | 0.81 |  | 0.79 | 1.00 | 0.99 | 0.91 |  | 0.86 | 0.89 | 0.93 | 0.88 |  | 0.79 | 1.00 | 0.89 | 0.91 |
| V7 | 0.93 | 0.38 | 0.67 | 0.93 |  | 0.93 | 0.50 | 0.90 | 0.70 |  | 0.86 | 0.63 | 0.89 | 0.73 |  | 0.86 | 0.81 | 0.90 | 0.83 |
| V8 | 0.71 | 0.59 | 0.68 | 0.65 |  | 0.79 | 1.00 | 0.86 | 0.90 |  | 0.86 | 0.88 | 0.87 | 0.87 |  | 0.64 | 0.71 | 0.77 | 0.68 |
| V9 | 0.86 | 0.63 | 0.85 | 0.73 |  | 0.63 | 0.90 | 0.89 | 0.79 |  | 0.71 | 0.84 | 0.91 | 0.79 |  | 0.71 | 0.68 | 0.83 | 0.70 |
| V10 | 0.86 | 0.68 | 0.85 | 0.76 |  | 1.00 | 1.00 | 1.00 | 1.00 |  | 1.00 | 0.90 | 1.00 | 0.94 |  | 0.86 | 0.68 | 0.80 | 0.76 |
|  |  |  |  |  |  |  |  |  |  |  |  |  |  |  |  |  |  |  |  |
| Mean | 0.84 | 0.68 | 0.83 | 0.78 |  | 0.74 | 0.92 | 0.91 | 0.84 |  | 0.84 | 0.88 | 0.92 | 0.86 |  | 0.81 | 0.82 | 0.86 | 0.82 |
| SD | 0.09 | 0.16 | 0.09 | 0.09 |  | 0.22 | 0.16 | 0.06 | 0.11 |  | 0.13 | 0.11 | 0.04 | 0.07 |  | 0.10 | 0.12 | 0.06 | 0.08 |
| CI | 0.05 | 0.10 | 0.05 | 0.05 |  | 0.14 | 0.10 | 0.04 | 0.07 |  | 0.08 | 0.07 | 0.02 | 0.04 |  | 0.06 | 0.07 | 0.04 | 0.05 |
| CV | 0.10 | 0.24 | 0.11 | 0.11 |  | 0.30 | 0.17 | 0.07 | 0.13 |  | 0.15 | 0.12 | 0.04 | 0.08 |  | 0.13 | 0.14 | 0.07 | 0.10 |
